# Substance use and duration of untreated psychosis in KwaZulu-Natal, South Africa

**DOI:** 10.4102/sajpsychiatry.v22i1.852

**Published:** 2016-05-20

**Authors:** Glen P. Davis, Andrew Tomita, Joy Noel Baumgartner, Sisanda Mtshemla, Siphumelele Nene, Howard King, Ezra Susser, Jonathan K. Burns

**Affiliations:** 1 Columbia University Medical Center, Department of Psychiatry, New York, NY, United States of America; 2 Columbia University Mailman School of Public Health, Department of Epidemiology, New York, NY, United States of America; 3 University of KwaZulu-Natal, Nelson R. Mandela School of Medicine, Durban, South Africa; 4 Duke Global Health Institute, Duke University, Durham, NC, United States of America; 5 KwaZulu-Natal Department of Health, Durban, South Africa; 6 Health Economics and HIV/AIDS Research Division (HEARD), University of KwaZulu-Natal, Durban, South Africa; 7 New York State Psychiatric Institute, United States of America

## Abstract

**Background:**

Substance use and psychiatric disorders cause significant burden of disease in low- and middle-income countries. Co-morbid psychopathology and longer duration of untreated psychosis (DUP) can negatively affect treatment outcomes.

**Objectives:**

The study assessed substance use amongst adults with severe mental illness receiving services at a regional psychiatric hospital in KwaZulu-Natal (South Africa). We describe the prevalence and correlates of lifetime substance use and examine the association between substance use and DUP.

**Methods:**

A cross-sectional survey recruited adults diagnosed with severe mental illness and assessed lifetime and past 3-month substance use using the World Health Organization Alcohol, Smoking and Substance Involvement Screening Test. Regression analyses were conducted to determine associations between lifetime substance use (other than alcohol and tobacco) and DUP as measured by the World Health Organization Encounter Form.

**Results:**

Amongst 87 participants, alcohol (81.6%), tobacco (75.6%) and cannabis (49.4%) were the most common substances reported for lifetime use. Risk of health-related problems (health, social, financial, legal and relationship) of cannabis use was associated with younger age, single marital status and lower education. Adjusted regression analyses indicated that use of amphetamines and methaqualone is associated with longer DUP.

**Conclusions:**

Substance use is prevalent amongst psychiatric patients in KwaZulu-Natal and may contribute to longer DUP. Mental health services in this region should address co-morbid substance use and psychiatric disorders.

## Introduction

Substance use and co-morbid psychiatric disorders contribute significantly to the global burden of disease.^1^ Co-occurrence of these disorders is common, and studies have shown that substance use is associated with poorer treatment outcomes of psychotic disorders.^2^

Another predictor of poorer outcomes for psychotic disorders is the duration of untreated psychosis (DUP), or the period of treatment delay between onset of psychotic symptoms and treatment initiation.^3^ DUP has been associated with greater negative symptom severity, greater functional impairment, and a more chronic long-term course of the disorder.^4^ In addition to its effect on clinical outcomes, longer DUP has been shown to be associated with reduced social functioning independent of symptoms,^5^ higher social disability,^6^ and reduced quality of life of affected patients.^7^

Although substance use and DUP both predict poorer outcomes for psychotic illness, the impact of substance use on DUP has not been clearly elucidated, particularly in South Africa where the prevalence of substance-use disorders is so high.^8^

Historically, substance use in South Africa was limited to locally produced alcohol, tobacco and cannabis.^8^ Over the past 20 years, the confluence of political, economic and social transformations of the post-apartheid era have been accompanied by an increase in South Africa’s vulnerability to substance use through increased availability and diversity of illicit drugs: Rapid modernisation, societal liberalisation, weakened border control and expansion of international trade have led to an escalation of drug trafficking and abuse.^8,9^

Cross-sectional surveys have documented co-morbid substance use and psychiatric disorders in the Western Cape (WC) Province of South Africa. A study of 95 young adult substance users in three inpatient substance-use treatment centres in Cape Town examined the frequency and nature of co-morbid psychopathology. The most common first substances used were cannabis (51.6%) and crystal methamphetamine (17.9%), and the most common co-morbid psychopathologies were anti-social personality disorder (87.4%) and conduct disorder (67.4%).^10^ Another survey of 298 inpatients at a psychiatric hospital in the WC showed that co-morbid substance-use disorder was diagnosed in 51%. Amongst the 8% diagnosed with a substance-induced psychiatric disorder, 1% was diagnosed with a substance-induced mood disorder and 7% with a substance-induced psychotic disorder.^11^

Whilst these studies provide insight into prevalence rates of substance use amongst psychiatric patients in the WC Province, they are not generalisable to other regions of South Africa, which differ ethnically and socio-economically from the rest of the country in ways that may influence substance-use trends.^12^

There are very few studies of substance use amongst psychiatric patients in KwaZulu-Natal (KZN) Province, a particularly vulnerable region to substance-related problems because of its high incidence of HIV and AIDS, unemployment and poverty compared to the national average.^13,14^ The objective of the current study is to describe substance-use patterns amongst patients at a regional psychiatric hospital in KZN, South Africa. Specifically, we sought to determine the type of substances used by patients with severe mental illness and describe the demographic risk factors for substance use amongst psychiatric patients. We further sought to examine substance use as a potential factor predicting the DUP.

## Methods

### Participants

This cross-sectional descriptive study of substance use surveyed individuals diagnosed with severe mental illness undergoing treatment at a provincial psychiatric hospital in Pietermaritzburg, South Africa, a regional referral psychiatric hospital in KZN. All consecutive individuals admitted into inpatient services (July 2012 to October 2013) who met study criteria were approached and invited to participate in the study. The inclusion criteria were DSM-IV-TR diagnosis of schizophrenia, schizoaffective disorder, bipolar disorder or psychotic disorder NOS and age > 21 years old. The exclusion criteria were determination of a developmental disability or cognitive impairment that would interfere with capacity to consent to participation. Those meeting the inclusion and exclusion criteria were approached and invited to participate. Each participant provided written informed consent after the study was explained in his or her first language. Of those eligible to participate in the sample, 97.8% gave their consent. The Biomedical Research Ethics Committee of the two participating institutions (University of KwaZulu-Natal and Columbia University) approved the study.

### Measurements

Each participant was administered a demographic survey to obtain various sociodemographic details. To measure substance use, the World Health Organization (WHO) Alcohol, Smoking and Substance Involvement Screening Test (ASSIST) was administered. This is a validated screening instrument for lifetime and recent (past 3 months) psychoactive substance users to establish numbers of substances and degrees of substance use and dependence.^15^ The ASSIST was developed for WHO by an international group of substance-abuse researchers for field testing in representative member states across the world. The instrument consists of eight questions with an administration time of approximately 10 minutes.

The first question asks about lifetime use of commonly used substances within the following 10 categories: tobacco, alcohol, cannabis, cocaine, amphetamine-type stimulants, inhalants, sedatives, hallucinogens and opioids. If a respondent reports no lifetime substance use for any of these substances, then the interview is terminated. If a respondent endorsed lifetime use of one or more substances, then Questions 2–5 ask are asked about recent drug use (during the ‘past three months’) for each substance category (frequency of use; cravings to use; frequency of health, social, legal or financial problems related to use). These questions are rated on a five-point frequency scale ranging from ‘never’ (in the past 3 months) to ‘daily’. Question 6 and Question 7 inquire about problems experienced with regard to lifetime substance use (concern expressed by family members regarding the subject’s substance use; failed attempts to control substance use; failure to meet expected obligations because of use), and Question 8 inquires about lifetime intravenous drug use.^15^

A completed ASSIST interview yields a total score, which is based on the sum of scores from Questions 2–7 (except for tobacco, which excludes Question 5 from scoring). After the total score is obtained, it is assigned a risk category, indicating the risk for problematic health consequences of lifetime and recent (past 3 months) use for each substance queried (for alcohol: 0–10 – Low; 11–26 = Moderate, 27+ = High; for other substances: 0–3 = Low; 4–26 = Moderate, 27+ = High).

As local terms for some psychoactive substances in South Africa are relatively unique,^9^ we modified the ASSIST by including local drug names in some categories (‘dagga’ under cannabis; ‘tik’ and ‘khat’ for methamphetamine and metacathinone, respectively, under amphetamine-type stimulants). We also asked the standard eight questions for the ASSIST to query polydrug mixtures that seem unique to South Africa. These include nyaope,^16^ and whoonga,^17,18,19,20^ two terms that have sometimes been used interchangeably to describe a mixture of substances including cannabis, low-grade heroin and crushed anti-retroviral (ARV) medications;^21^ sugars, a term used to describe heroin mixed with cocaine;^22^ Mandrax (also known as methaqualone) and dagga combined with Mandrax, colloquially known as ‘white pipe’.^23^

Duration of untreated psychosis was assessed using the Encounter Form,^24^ an instrument developed by WHO and used in many sub-Saharan African countries to obtain information on participant characteristics and sources of care before reaching the mental health service. The Encounter Form queries the DUP by asking the number of months between the participant’s first symptom and the date when he or she was first seen by clinical or formal mental health services. The DSM-IV-TR psychiatric diagnoses were based on clinical interviews by psychiatrists on the inpatient unit at a provincial psychiatric hospital.

Trained research assistants administered the study instruments, which were translated from English to isiZulu and back-translated according to WHO guidelines using an expert panel of reviewers to verify accuracy and cultural sensitivity of the translations. isiZulu-speaking research assistants were trained to administer the instruments, and the interviews were administered in each participant’s first language (isiZulu or English).

### Data collection, statistical methods and analysis

The data were collected from July 2012 to October 2013 and entered and analysed using the Statistical Package for Social Sciences version 21 and STATA 13. Frequency analysis was used to determine (lifetime and past 3 months) occurrence of substances used in the sample. ASSIST raw scores and risk levels were calculated as described above and as standard procedure for the ASSIST. Bivariate analysis was conducted to determine associations between ASSIST scores for cannabis use and DUP as well as other demographic characteristics. Poisson regression analyses were conducted for lifetime use of each substance listed in the ASSIST, with DUP as the main predictor variable. Both unadjusted and adjusted regression models are presented.

## Results

Table 1 provides demographic and clinical characteristics of the study sample (*N* = 87; 27 outpatients, 60 inpatients). Participants were mostly men (56.3%), black South African (64.4%), aged 21–29 years (40.2%) and educated beyond grade 12 (56.3%). Most participants described themselves as living in an urban environment (59.8%) and had a broad distribution of marital status (married/stable partner 34.9%; casual partner 23.3%; no relationship or partner 41.9%). The most frequent psychiatric diagnosis represented in the sample was schizophrenia (41.4%), followed by bipolar disorder (32.2%) and schizoaffective disorder (16.1%).

**TABLE 1:** Demographic and clinical characteristics (*N* = 87).

Variable	*n*	%
**Gender**		
Male	49	56.3
Female	38	43.7
**Age category**		
21–29	35	40.2
30–39	25	28.7
40+	27	31.0
**Education**		
< Grade 12 or equivalent	38	43.7
≥ Grade 12 or equivalent	49	56.3
**Race or ethnicity**		
Black	56	64.4
Non-Black†	31	35.6
**Residence**		
Urban	52	59.8
Rural	35	40.2
**Marital status**		
Married/stable partner	30	34.9
Casual partner	20	23.3
No relationship or partner	36	41.9
**Psychiatric diagnosis**		
Schizophrenia	36	41.4
Schizoaffective	14	16.1
Bipolar disorder	28	32.2
Psychosis NOS and other	9	10.3

† Non-Black refers to White people or Mixed-race people.

Table 2 describes the lifetime substance use and the ASSIST risk score for each substance queried. For lifetime use, the substances most commonly used were alcohol (81.6%), tobacco (75.6%) and cannabis or dagga (49.4%). Lifetime use of cocaine (4.6%) and nyaope or sugars (2%) were reported least frequently. For each substance, an ASSIST risk category was assigned (low, medium or high), indicating a risk for problematic health consequences because of using that substance (health, social, financial, legal and relationship problems). The ASSIST risk category for each substance was based on questions related to both lifetime and recent (past 3 months) use of that substance (Questions 2–5 ask about past 3 months use; Question 6 and Question 7 ask about lifetime use; risk score is derived from summation of responses to Questions 2–7). For almost all substances, including those reported infrequently, most participants scored in the moderate range (4–26) for risk of health-related problems. For alcohol, 62% were scored as low risk, 32.4% as moderate risk and 5.6% as high risk. This trend was similar for cannabis or dagga (53.5% low risk, 39.5% moderate risk and 6.9% high risk). Although use of hallucinogens was only reported by 5.8% of the sample, 20% of these reported high risk of problematic hallucinogen use. Only two participants in the sample reported a lifetime history of injection drug use.

**TABLE 2:** Substance-use characteristics.

Variable	ASSIST		ASSIST (raw score)				ASSIST risk level (%)		
		
	Lifetime use		Lifetime and recent use				Lifetime and recent use		
		
	*n*	%	Mean score	SD	Median score	IQR	Low (0–3)	Moderate (4–26)	High (27+)
Tobacco products (cigarettes, chewing tobacco, cigars, etc.)	65	75.6	15.1	8.5	18.0	9.0	16.9	76.9	6.2
Alcoholic beverages (beer, wine, spirits, etc.)	71	81.6	8.8	9.4	6.0	16.0	62.0	32.4	5.6
Cannabis (dagga, marijuana, etc.)	43	49.4	8.7	10.2	3.0	17.0	53.5	39.5	6.9
Cocaine (coke, crack, etc.)	4	4.6	5.0	7.1	2.5	10.0	50.0	50.0	0.0
Amphetamine-type stimulants (tik, metacathinone or ‘khat’, speed, diet pills, ecstasy, etc.)	11	12.6	7.0	7.8	4.0	17.0	45.5	54.6	0.0
Inhalants (nitrous, glue, petrol, paint thinner, etc.)	8	9.2	3.9	5.1	1.5	7.0	62.5	37.5	0.0
Sedatives or sleeping pills (Valium, Serepax, Rohypnol, etc.)	14	16.1	10.4	10.7	11.5	17.0	42.9	50.0	7.1
Hallucinogens (LSD, acid, mushrooms, PCP, Special K, etc.)	5	5.8	15.0	28.6	3.0	6.0	60.0	20.0	20.0
Opioids (heroin, pills for headache or other chronic pain, such as pethidine, morphine, codeine, grandpas or others)	6	7.0	16.8	8.6	19.0	5.0	16.7	83.3	0.0
Nyaope/sugars	2	2.3	6.5	4.9	6.5	7.0	50.0	50.0	0.0
Whoonga	5	5.75	10.8	9.9	5.0	17.0	40.0	60.0	0.0
Mandrax (dagga + Mandrax or ‘white pipe’)	9	10.34	6.8	7.6	3.0	11.0	55.6	44.4	0.0

ASSIST, Alcohol, Smoking and Substance Involvement Screening Test; LSD, Lysergic acid diethylamide; PCP, Phencyclidine; SD, standard deviation.

Same sample size (*n*) across lifetime, ASSIST raw score and risk-level analysis per substance-use category. For alcohol only: 0–10 – Low; 11–26 = Moderate; 27+ = High.

For the three most commonly used substances, recent (past 3 months) use was 88% (tobacco), 54% (alcohol) and 43% (cannabis/dagga). Use of multiple substances was also reported amongst the sample, with 30 unique combinations of lifetime substances used. The most commonly reported combinations were tobacco + alcohol + cannabis or dagga (*n* = 22; 25%) and tobacco + alcohol (*n* = 16; 18%). Only 10% of participants reported no lifetime history of any substance use.

Owing to the frequency of cannabis or dagga use relative to other non-alcohol substance use, we were interested in examining the associated demographic and clinical characteristics associated with cannabis or dagga (Table 3). There was a significant correlation between higher ASSIST scores for cannabis or dagga use (and thus high risk of problematic use) and younger age (21–29 years), lower level of education and single marital status. No significant correlation was observed with regard to gender, ethnicity, residence or psychiatric diagnosis.

**TABLE 3:** Bivariate analysis between ASSIST risk score (lifetime and recent cannabis use) and demographic/clinical characteristics (*n* = 87).

Variable	Characteristic	ASSIST – Cannabis							Statistics

		Low	Mod	High	Statistics	Mean score	Statistics	Median score	
**Gender**	-	-	-	-	*χ*^2^ = 3.7, *p* = 0.15	-	*t* = −0.6, *p* = 0.56	-	*z* = −0.7, *p* = 0.49
	Male	20	11	3	-	8.2	-	3	-
	Female	3	6	0	-	10.4	-	9	-
**Age**	-	-	-	-	*χ*^2^ = 8.3, *p* = 0.08		*F* = 2.6, *p* = 0.09	-	*χ_a_*^2^ = 7.2, *p* = 0.03
	21–29	10	12	3	-	11.2	-	9	-
	30–39	7	5	0	-	7.0	-	1.5	-
	40+	6	0	0	-	1.5	-	1.5	-
**Education**	-				*χ*^2^ = 6.7, *p* = 0.04		*t* = 1.9, *p* = 0.06		*z* = 2.2, *p* = 0.03
	< Grade 12	7	12	2		11.7		10	
	≥ Grade 12	16	5	1		5.8		0	
**Race/ethnicity**	-	-	-	-	*χ*^2^ = 2.5, *p* = 0.28	-	*t* = 1.3, *p* = 0.20		*z* = 1.1, *p* = 0.26
	Black	14	13	3		10.0		8.5	-
	Non-black	9	4	0		5.6		2	-
**Marital status**	-	-	-	-	*χ*^2^ = 16.9, *p* < 0.01	-	*F* = 7.2, *p* < 0.01	-	*χ_a_*^2^ = 13.4, *p* < 0.01
	Married/stable partner	5	8	0	-	9.5	-	8	-
	Casual partner	3	7	2	-	14.3	-	13	-
	No relationship/partner	15	2	0	-	2.6	-	0	-
**Residence**		-	-	-	*χ*^2^ = 0.9, *p* = 0.63		*t* = −0.6, *p* = 0.56		*z* = −0.6, *p* = 0.55
	Urban	14	8	2		7.8		3	
	Rural	9	9	1		9.7		10	
**Psychiatric diagnosis**	-	-	-	-	*χ*^2^ = 7.3, *p* = 0.29		*F* = 1.4, *p* = 0.26	-	*χ_a_*^2^ = 5.3, *p* = 0.15
	Schizophrenia	16	6	2	-	7.1	-	1	-
	Schizoaffective	1	4	1	-	16.3	-	15	-
	Bipolar	4	5	0	-	7.7	-	6	-
	Psychosis NOS and other	2	2	0	-	8.5	-	7	-

ASSIST, Alcohol, Smoking and Substance Involvement Screening Test.

*χ*_a_^2^ based on median test. *Z* is based on rank-sum test.

Table 4 presents the associations between the ASSIST score for each substance and the DUP. In unadjusted regression analysis, lifetime use of cannabis, amphetamine, hallucinogens, whoonga and Mandrax was associated with longer DUP. When adjusted for sociodemographic characteristics, multiple regression analysis revealed significant association between use of amphetamine and Mandrax with longer DUP. Irrespective of substance use, the following characteristics were commonly associated with longer DUP: young age (21–29 years), completion of high school, having a partner (married/stable partner) and no income.

**TABLE 4:** Unadjusted and adjusted regression analysis between Alcohol, Smoking and Substance Involvement Screening Test and duration of untreated psychosis (DUP) for lifetime substance use.

Variable	Main predictor – unadjusted				Main predictor – adjusted				Adjusted model – significant covariates for longer DUP
	
	*β*	SE	*Z*	*p*	*β*	SE	*Z*	*p*	
Tobacco products	0.17	0.09	1.86	0.06	0.21	0.12	1.79	0.07	Stable partner/married/casual partner, high school graduate
Alcoholic beverages	0.16	0.10	1.61	0.11	0.15	0.13	1.17	0.24	Stable partner/married/casual partner, no income, high school graduate
Cannabis	0.15	0.07	2.28	0.02	0.04	0.09	0.40	0.69	Stable partner/married/casual partner, high school graduate
Cocaine	0.26	0.09	2.96	< 0.01	0.08	0.11	0.76	0.45	Stable partner/married/casual partner, no income, young age, high school graduate
Amphetamine-type stimulants	0.33	0.07	4.73	< 0.01	0.26	0.10	2.61	0.01	Stable partner/married, no income
Inhalants	0.07	0.09	0.81	0.42	0.04	0.11	0.40	0.69	Stable partner/married/casual partner, young age, high school graduate
Sedatives or sleeping pills	0.08	0.09	0.98	0.33	0.05	0.09	0.52	0.60	Stable partner/married/casual partner, young age, high school graduate
Hallucinogens	0.19	0.09	2.07	0.04	0.09	0.12	0.76	0.44	Stable partner/married/casual partner, young age, high school graduate
Opioids	−0.60	0.56	−1.07	0.29	−0.44	0.58	−0.77	0.44	Stable partner/married/casual partner, young age, high school graduate
Nyaope/sugars	−0.35	0.27	−1.28	0.20	−0.19	0.29	−0.66	0.51	Stable partner/married/casual partner, high school graduate
Whoonga	0.20	0.09	2.32	0.02	0.10	0.11	0.92	0.36	Stable partner/married, high school graduate
Mandrax	0.29	0.07	4.29	< 0.01	0.27	0.10	2.84	< 0.01	Stable partner/married, no income

## Discussion

The high percentage of individuals diagnosed with serious and persistent mental illness who endorsed the use of psychoactive substances (particularly alcohol and cannabis or dagga) reflects the growing trend of substance use in South Africa and indicates frequent occurrence of psychiatric comorbidity in KZN. The rate of cannabis or dagga use was similar to previous studies of substance use amongst psychiatric patients in the WC,^10,11^ although those studies reported a higher prevalence of methamphetamine use. The rates of substance use in the current study were significantly higher than those reported in surveys of the general population in South Africa^8,25,26^ highlighting the need for proactive inquiry about substance use amongst both inpatients and outpatients of psychiatric services in the province. Notably, a study performed by Burns and colleagues found that the prevalence of recent (past month) use of cannabis was 35% amongst participants with first-episode psychosis.^27^ In the current study, past 3-month cannabis use (43%) cannot be directly compared to the past-month cannabis-use prevalence from the Burns *et al*. study, but both figures collectively indicate common frequency of recent cannabis use.

Overall, the degree of substance-related health problems and risk of dependence (as measured by ASSIST risk score) was lower than expected across all substances with a small percentage of ASSIST risk scores in the high range, with the exception of hallucinogens (20% high risk). However, the fact that most ASSIST risk scores fell in the moderate range indicates some degree of acknowledgement of problematic substance use amongst the sample. Substance users often misreport their frequency and pattern of use because of social stigma and criminal sanctions. Denial is a common response style amongst substance-abusing populations, and the consequences of substance use are often minimised.^28^ In a clinical setting, obtaining collateral history from family members and other primary supports might reflect a higher degree of impairment amongst substance-using psychiatric patients than those suggested by the ASSIST risk scores in the study.

The data suggest an association between use of amphetamine or Mandrax and longer DUP. In the case of both methamphetamine and Mandrax, which can induce psychosis in individuals not otherwise diagnosed with mental illness,^29^ one explanation for the longer DUP is that experienced users of these substances may be accustomed to sub-syndromal psychotic symptoms, which they dismiss as closely related to substance use. Therefore, such individuals might resist formal psychiatric care until symptoms become pervasively debilitating, at which time they or their family members recognise the need for psychiatric treatment.

Notably, longer DUP was associated with cannabis use in the bivariate analysis (although the association lost significance in the regression), in contrast to a previous study amongst psychiatric patients at the same hospital in KZN conducted by Burns and colleagues, which showed that cannabis use was associated with shorter DUP.^27^ An important difference between these two studies is that the Burns *et al*. study defined cannabis use in terms of recent (past month) use, whereas the current study assessed DUP against lifetime cannabis use. It may be that the timing of cannabis use is important in terms of how this exposure impacts on DUP.

Burns published a systematic review and meta-analysis examining cannabis use and DUP.^30^ Whilst the review did not find a significant relationship between cannabis use and DUP, the author noted that studies examining lifetime cannabis use were associated with longer DUP, whilst those examining recent cannabis use were associated with shorter DUP. Regarding why the pattern of cannabis use (lifetime versus recent) might affect DUP differently, Burns cited evidence that lifetime (early, long-term) cannabis use may be associated with neurodevelopmental impairment and negative symptoms at psychosis onset, often presenting with an insidious course, delayed treatment seeking and poorer outcome.^30^ Current or recent exposure to cannabis may be associated with a more acute illness, characterised by florid positive and behavioural symptoms, possibly hastening the pathway to care and reflecting a shorter DUP.^30^

Whoonga, a loosely defined term for a polydrug mixture that may include cannabis, heroin and crushed antiretroviral medications^17,18,19,20,21^ was not frequently reported to be used by participants in this sample. Grelotti and colleagues have reported original, qualitative data from semi-structured interviews indicating that whoonga may be an emerging drug of abuse in urban areas of South Africa^18,19^, a concerning phenomenon, given that alleged use of diverted antiretroviral (ARV) medications as an ingredient may undermine adherence to HIV treatment and perpetuate ARV resistance^17^. The relatively infrequent use of whoonga reported in this sample is reassuring, but cautiously so, given the relatively small sample size. In light of potential public health ramifications of ARV diversion and recreational use^17,18,19,20,21^, further research on the prevalence of whoonga and similar polydrug mixtures in South Africa remains warranted.

A number of limitations of the current study are acknowledged. Firstly, sample size was small, and the cross-sectional method did not enable causative factors to be identified with regard to substance use. All information on substance use relied on self-report, which may have compromised the reliability of the information resulting in under- or over-reporting of substance use. Participants’ reported substance use was not verified by biomarkers or collateral information from family. The study only included participants who interfaced with the formal mental health system and therefore does not reflect preponderance of substance use amongst individuals with mental illness who may seek help through the highly used system of traditional practitioners in KZN or through other informal and formal service providers (e.g. general district hospitals). Furthermore, the sample is derived from a hospitalised patient population, which, within the local context, is a select population of acutely mentally ill patients. Many of them may have accessed care owing to the socially disruptive nature of their illness, and the select nature of this sample may have impacted on our results. However, it would be speculative to debate the directionality of this effect.

The current study highlights that substance use is prevalent amongst individuals receiving psychiatric treatment in KZN Province. It is important for general psychiatric clinicians to inquire about substance use amongst patients who present with symptoms of severe and persistent mental illness and for appropriate treatment services and mental health programs to be designed to address the complex needs in this underserved region.
